# Validity and Safety of Robot-Assisted Laparoscopic Radical Cystectomy for the Elderly: Results of Perioperative Outcomes in Patients Aged ≥80 Years

**DOI:** 10.5152/tud.2022.22099

**Published:** 2022-09-01

**Authors:** Kenji Tanabe, Yasukazu Nakanishi, Yosuke Umino, Naoya Okubo, Madoka Kataoka, Shugo Yajima, Hitoshi Masuda

**Affiliations:** 1 Department of Urology, National Cancer Center Hospital East, Chiba, Japan

**Keywords:** Urinary bladder neoplasms, aged, 80 and over, postoperative complications, cystectomy, robotic surgical procedures

## Abstract

**Objective:**

To improve perioperative outcomes, robot-assisted radical cystectomy has gained increasing interest. This study aimed to assess the detailed perioperative complications of robot-assisted radical cystectomy in elderly aged ≥80 years and compare them with those of non-elderly.

**Material and methods:**

We retrospectively analyzed the clinical features of 74 patients who underwent robot-assisted radical cystectomy for bladder cancer between September 2018 and September 2021. Perioperative complication was classified by the Clavien–Dindo classification and organ system-based categories. We assessed the relationship between age or Charlson comorbidity index score (≥3 or <3) and the incidence of perioperative complication or rehospitalization rate within 90 days postoperatively.

**Results:**

Of the 74 patients, perioperative complication of all grades and grade ≥IIIa occurred in 54 (73%) and 15 (20%) patients, respectively. The postoperative rehospitalization rate was 20%, and the perioperative mortality rate was 0%. Elderly (n = 20) showed no difference in the incidence of perioperative complication of all grades or grade ≥IIIa compared with non-elderly, and no organ system-based category had a higher incidence in elderly than that in non-elderly. Gastrointestinal tract-related perioperative complication incidence was higher in non-elderly and those with Charlson comorbidity index ≥3 (*P* = .044, .039, respectively); cardiovascular-related perioperative complication incidence was higher in those with Charlson comorbidity index ≥ 3 (*P* = .0068).

**Conclusion:**

The incidence perioperative complication of robot-assisted radical cystectomy in elderly was not different from those in non-elderly, suggesting that robot-assisted radical cystectomy may be an option for the treatment of bladder cancer in elderly as well as non-elderly.

**Main Points**

- This study aimed to assess the detailed perioperative complications of robot-assisted radical cystectomy in elderly aged ≥80 years and compare them with those of non-elderly.
- The incidence of perioperative complications of robot-assisted radical cystectomy in elderly aged ≥80 years was not different from those in non-elderly.
- Robot-assisted radical cystectomy may be an option for the treatment of bladder cancer in the elderly as well as non-elderly.

## Introduction

The incidence of bladder cancer increases proportionally with age, with approximately 1.7 cases per 1000 individuals aged ≥80 years in Japan. As the world population ages, the number of patients with bladder cancer, especially the elderly, is increasing.^1^ Therefore, the demand for the treatment of bladder cancer is also increasing. Radical cystectomy (RC) is a curative treatment for localized muscle-invasive bladder cancer (MIBC) and high-risk non-muscle-invasive bladder cancer (NMIBC) and is one of the major surgical procedures in urology^2^; however, it is associated with high perioperative complications (PCs) and perioperative mortality due to its high surgical invasiveness.^3^ Particularly, the risk of perioperative mortality is high in the elderly population aged ≥80 years, and indications for surgery are difficult to determine in this age group.^3,4^

Recently, with the widespread use of robot-assisted laparoscopic RC (RARC), a minimally invasive surgery^5^ with a cancer control ability similar to that of open RC (ORC),^6,7^ it is expected that RC can be safely performed on the elderly. Some reports showed no difference in the PC of RARC in the elderly compared with that of non-elderly.^8^ With the increasing demand for RARC in the elderly population, more detailed PCs are desirable.

In this study, we analyzed the detailed PC of RARC in elderly and compared them with those of non-elderly.

## Materials and Methods

### Patients

We retrospectively analyzed the clinical data of 74 consecutive patients who underwent RARC for localized MIBC and high-risk NMIBC at our institution between September 2018 and September 2021. All 74 cases were followed up for >90 days. Of the 74 patients, we defined the patients aged ≥80 years as elderly (n = 20) and the rest as non-elderly (n = 54). All patients underwent transurethral resection of bladder tumor prior to RC and were diagnosed with bladder cancer. Cisplatin-based chemotherapy was administered as a preoperative treatment for patients with MIBC. Before surgery, all patients underwent electrocardiography and echocardiography and subsequently visited a cardiologist for evaluation of their surgical tolerance. This retrospective study was approved by the National Cancer Center Research Ethics Review Committee (Research Project No. 2018-159). and complied with the principles of the Declaration of Helsinki. All patients provided wirtten informed consent preoperatively.

### Surgical Procedure

Patients underwent RARC with bilateral pelvic lymph node resection as far as possible to the level of the common iliac artery, followed by urinary diversion. The procedure was performed by 3 surgeons, 2 of whom were novice surgeons with <20 RARCs. The 2 surgeons performed the procedure under the guidance of a senior surgeon. Urinary diversion was performed by ileal conduit (IC), ileal neobladder, or ureterocutaneostomy. The IC was created by the conventional extracorporeal urinary diversion (ECUD) until May 2020 and intracorporeal urinary diversion (ICUD) thereafter. The resection lengths of the terminal ileum were 20 and 55 cm for the IC and neobladder, respectively. Even in patients who underwent ICUD, intestinal anastomosis was performed by functional end-to-end anastomosis combined with laparotomy through a small incision. Ureteral stents were placed at the time of urinary diversion.

### Perioperative Management

All patients were on the same clinical pathway. Patients consumed normal meals orally until the day before surgery and took stimulant laxatives orally the night before surgery. Intraoperatively, all patients received prophylactic antimicrobial agents (second-generation cephalosporins). Blood transfusions were administered for intraoperative and postoperative acute anemia. Ambulation was encouraged as much as possible on the first postoperative day. Patients began drinking water on the first postoperative day, and the physician in charge determined diet based on the patient’s general condition and abdominal x-ray findings. Ureteral stents were removed or replaced one at a time approximately 2 weeks postoperatively. The day of discharge was decided by the physician in charge based on the patient’s general condition.

### Data Collection

The following variables were analyzed: age, gender, body mass index (BMI), Eastern Cooperative Oncology Group performance status (ECOG PS), American Society of Anesthesiology physical status, Charlson comorbidity index (CCI),^9^ history of smoking, abdominal surgery, and abdominal radiotherapy, clinical staging, presence of neoadjuvant chemotherapy, preoperative anemia, and preoperative renal dysfunction, urine diversion method, operative time, the estimated blood loss (EBL), presence of perioperative blood transfusion, and pathological staging. Charlson comorbidity index was classified as 0-2 and ≥3.^10^ The eighth edition of the International Union Against Cancer was used for staging. Blood samples were collected approximately 1 month before the date of surgery and the day before surgery. Anemia was defined as hemoglobin levels of <13.5 and <12.0 g/dL in males and females, respectively.^11^ Renal dysfunction was defined as an estimated glomerular filtration rate of <60 mL/min/1.73 m^2^.^12^

### Perioperative Outcomes

Perioperative outcomes included the incidence of PC, rehospitalization within 90 days postoperatively, and mortality within 90 days postoperatively. Perioperative complications were defined as undesirable medical events occurring before hospital discharge. All PCs were graded according to the Clavien–Dindo classification (CDC) and classified as grades ≥IIIa and <IIIa. Moreover, all PCs were classified by organ system-based categories.

### Statistical Analysis

We assessed the relationship between age or CCI score and perioperative outcomes. Differences between groups were analyzed using chi-square test or Fisher’s exact test for categorical variables and the Wilcoxon–Mann–Whitney *U*-test for continuous data. Hypothesis testing for the difference in the population proportions was used to evaluate the association between age or CCI score and the incidence of PC or the rehospitalization rate within 90 days postoperatively.

As a subgroup analysis, a similar analysis was performed between the ICUD and ECUD groups in patients who underwent IC.

Statistical significance was set at 2-tailed *P* values <.05. The statistical analysis methods were determined after consultation with the hospital’s statistics department. All statistical analyses were performed using the JMP software (SAS Institute Inc., version 13.2).

### Ethical Standards and Policies

The study protocol was approved by the institution’s Ethics Committee and complied with the provisions of the Declaration of Helsinki (National Cancer Center Research Ethics Review Committee, Research Project No. 2018-159). All patients provided informed consent.

## Results

Table 1 shows the clinical, operative, and pathological characteristics of the elderly and non-elderly patients, respectively. The overall median (range) age was 74 (39–89) years, and 60 (81%) patients were male. A total of 65 (88%) patients had a CCI of 0-2 and 9 (12%) had ≥3. A total of 63 (85%) patients had a history of smoking, 26 (35%) had a history of abdominal surgery, and 1 (1%) had a history of abdominal radiotherapy. A total of 37 (50%) patients received neoadjuvant chemotherapy. Elderly had higher ECOG PS (*P* = .0035), higher clinical N stage (*P* = .012), and shorter operative time (*P *= .0025) than non-elderly. There was no difference in pathological results between elderly and non-elderly.

Overall, PC of all grades and ≥IIIa in CDC occurred in 54 (73%) and 15 (20%) patients, respectively (Table 2). The highest incidence of PC in all grades in CDC was genitourinary PC (32%), followed by gastrointestinal tract- and bleeding-related PCs (27% and 22%, respectively). Only 1 patient developed cardiovascular-related PC, and no patient developed respiratory-related PC.

Tables 3–5 present the perioperative outcomes by age or CCI score. Table 3 shows the incidence of PC of all grades, grade ≥IIIa, and grade <IIIa in CDC and the rehospitalization rate within 90 days postoperatively. For the entire cohort, PC of all grades in CDC occurred in 54 (73%) patients, and the rehospitalization rate was 20%. Perioperative complication of grades ≥IIIa and <IIIa in CDC occurred in 15 (20%) and 51 (69%) patients, respectively, and 12 (16%) patients developed both grades≥IIIa and <IIIa PC. No relationship between age or CCI score and perioperative outcomes was observed. Table 4 shows the incidence of PC of all grades in CDC by organ system-based categories. Non-elderly and those with CCI ≥3 had a higher incidence of gastrointestinal tract-related PCs (*P* = .044, *P* = .039, respectively), and the patients with CCI ≥3 had a higher incidence of cardiovascular-related PCs (*P* = .0068). Table 5 displays the incidence of PC of grade ≥IIIa in CDC according to the PC type. Intraoperative complications were the most common PC of grade ≥IIIa in CDC (7%), followed by postoperative ileus and ureteral stricture (5% and 4%, respectively). The group with CCI ≥3 had a higher incidence of cardiovascular-related PCs (*P* = .0068).

Table 6 shows the results of hypothesis testing for the difference in the population proportions between age or CCI score and PCs or postoperative rehospitalization rates within 90 days postoperatively. The patients with CCI ≥3 had higher population proportions of the incidence of all grade PCs than those with CCI <3 (*P* = .031). The elderly had lower population proportions of the incidence of gastrointestinal tract-related PCs of all grades in CDC than the non-elderly (*P* = .035). The population proportions of the incidence of cardiovascular-related PCs did not differ between the age and CCI score groups. Regarding the PC of grade ≥IIIa in CDC according to the PC type, there was no difference in their respective population proportions.

No deaths within 90 days postoperatively were noted in this cohort.

In a subgroup analysis of only patients who underwent IC, those who underwent ICUD had a smaller proportion of the elderly and a lower incidence of bleeding-related PCs of all grades in CDC (*P* = .027 and .041, respectively). The incidence of other PCs or the population proportions of any type of PCs did not differ between the ICUD and ECUD groups (Supplementary Table 1–4).

## Discussion

This study shows for the first time that RARC is safe for patients aged ≥80 years, especially in terms of no increase in PC. Noteworthy points are as follows: (1) No difference in the incidence of overall PC of all grades and grade ≥IIIa in CDC between elderly and non-elderly or between patients with CCI ≥3 and those with CCI <2 was noted, except that the incidence of PC of all grades in CDC was higher in patients with CCI ≥3. (2) No difference in the postoperative rehospitalization rate within 90 days postoperatively between elderly and non-elderly or between patients with CCI ≥3 and those with CCI <2 was observed. (3) No difference in the incidence of organ system-based PC for all grades in CDC between elderly and non-elderly or between patients with CCI ≥3 and those with CCI <2 was noted, except that the incidence of gastrointestinal tract-related PCs was higher in non-elderly. (4) No difference in the incidence of PC of grade ≥IIIa for events between elderly and non-elderly or between patients with CCI ≥3 and those with CCI <2 was observed.

In the case of ORC, the incidences of PC and perioperative mortality are increased in the elderly than in non-elderly.^3,13,14^ According to a systematic review that focused on the PC of RC, including 90% ORC, the incidence of PC in all grades and grade ≥IIIa in CDC was 58.5% and 16.9% in the elderly, respectively.^15^ Fairey et al^16^ showed that the incidence of PC after RC was 47% and 24% for all PCs and major PCs, respectively, and reported that the incidence of PC increased with the severity of comorbidities. In a large study of 1264 patients who were divided according to aged <80 and ≥80 years, the incidence of PC was higher in the elderly, with a predominance of cardiovascular-related PCs and a trend toward higher incidences of respiratory- and gastrointestinal tract-related PCs.^14^ Yamanaka et al^17^ reported in a study of 629 patients who underwent ORC that no difference was observed in the overall incidence of PC in patients aged ≥80 years compared with non-elderly; however, the incidence of urinary tract infection was higher. Therefore, the indications for RC for MIBC and high-risk NMIBC in the elderly have been carefully determined.

Robot-assisted radical cystectomy has been reported to have non-inferior cancer control ability^6,7^ and be less invasive than ORC, with less EBL and PC, and shorter hospital stay.^18^ In a systematic review using the Cochrane Database, Rai et al^19^ found that RARC and ORC may have similar outcomes with regard to time to recurrence, rates of major complications. Similar results were obtained when RARC was compared with ORC in a cohort of elderly only. Nguyen et al^8^ reported acceptable PC rates with 44% and 14% incidence of all grades and grade ≥IIIa PC in CDC, respectively, in patients aged ≥80 years who underwent RARC. Richards et al^20^ compared the incidence of PC in patients aged ≥75 years who underwent RARC and ORC and reported that the incidence of grade ≥IIIa PC in CDC was 10% in RARC, which was lower than that in ORC (35%). Both authors argue that these conclusions hold promise for expanding the indications for RC for elderly. However, there are few reports comparing the perioperative outcomes of elderly and non-elderly who underwent RARC, and no conclusions have been drawn as to whether the indications for RARC in elderly are the same as those in non-elderly. A report of 99 cases comparing PC of grade ≥IIIa in CDC between elderly (≥70 years old, n = 38) and non-elderly (<69 years old, n = 61) who underwent RARC showed no difference between the 2 groups (34% vs. 36%, respectively) and concluded that RARC may be an option for bladder cancer in both elderly and non-elderly.^21^ In this study, the incidence of grade ≥IIIa PC in CDC in 20% of elderly was almost the same as that reported in previous studies, and the results were comparable with those in non-elderly. Although the incidence of PC of all grades in CDC was higher than that reported by other authors, this means that there are several minor PCs in this study cohort, a common trend seen in reports of the incidence of post-RC PC in Japan.^22,23^ This may be due to the longer hospitalization period in the Japanese healthcare system than that in Western countries, which makes it easier to detect minor PCs.

Previous studies of PC by organ system-based categories reported a relatively high incidence of gastrointestinal tract-related and genitourinary PCs, with cardiovascular- and respiratory-related PCs also being common.^8,16,24^ In this study, the incidence of all grades in CDC of gastrointestinal tract-related and genitourinary PCs was also higher than that of other PCs at 27% and 32%, respectively. The chi square test showed that the incidence of gastrointestinal tract-related PCs tended to be higher in non-elderly and those with CCI ≥3 (*P* = .044 and .039, respectively); however, the difference in the population proportions was found only between ages. The incidence of cardiovascular- and respiratory-related PCs was significantly lower in this study than in previous ones.^8,16,24^ Regarding cardiovascular-related PCs, one factor may be that all patients underwent screening tests of cardiac function and a visit to a cardiologist before surgery to assess their ability to tolerate the procedure. Although no patient had to discontinue the surgery due to the results of the evaluation of surgical tolerance, 2 (2%) patients were started on anticoagulants owing to angina pectoris. Regarding respiratory-related PCs, the significance of prognostic nutritional index (PNI) with a cut-off value of 45 on respiratory-related PC in RC was recently reported.^25^ In this study, we did not find any respiratory-related PC. Preoperative spirometry showed ventilatory impairment in 21 patients. Of these patients, only 6 had a low PNI (<45), and even the 6 patients did not have a significant decrease in PNI with a mean value of 42.4 (individual data was not shown), suggesting that this may be the reason why respiratory tolerance was achieved.

In this study, PC of grade ≥IIIa in the CDC occurred in 20% of the patients overall, and the postoperative rehospitalization rate within 90 days was 20%. This was consistent with the RARC perioperative outcomes reported by other authors.^18,26^ The elderly group had a higher ECOG PS and shorter operative time than the non-elderly group. These results were consistent with those reported by other authors comparing elderly and non-elderly who underwent RC, including ORC.^3,24^ The absence of differences in the incidence of PC of grade ≥IIIa in CDC and postoperative rehospitalization rates between elderly and non-elderly in the cohort with no significant difference in clinical characteristics without ECOG PS, CCI, and clinical N stage between the 2 groups suggests that RARC may be a treatment option for bladder cancer in elderly and non-elderly. These results were generally similar in the subgroup analysis between the ICUD and ECUD groups in patients who had undergone IC only. The overall length of postoperative hospital stay was longer than that reported by other authors^3,26^; however, this was attributed to the unique Japanese practice system.

Our study has some limitations. First, this was a retrospective cohort study with a small number of 74 cases. In addition, the small number of patients older than 80 years old reduced our statistical power. We plan to accumulate more cases and examine the results, including those from other institutions, in the future. Second, our patients underwent a specialized preoperative evaluation of their surgical tolerance by a cardiologist, which may have been influenced by the results of cardiovascular-related PCs in particular. Finally, there were multiple surgeons with varying years of experience. However, all surgeons used the same technique with little variability at our institution, and we believe that evaluating the results of multiple surgeons is realistic.

In conclusion, perioperative outcomes of RARC in elderly were not different from those in non-elderly, suggesting that RARC may be an option for the treatment of localized MIBC and high-risk NMIBC in elderly aged ≥80 years as well as in non-elderly.

## Figures and Tables

**Table 1.** Clinical, Operative, and Pathological Characteristics of Patients in the Elderly and Non-Elderly Groups

Variable	Total (n = 74)	Elderly^*^ (n = 20)	Non-elderly^†^ (n = 54)	*P*
Age (years), median (range)	74 (38-89)	81 (80-89)	72 (39-79)	<.0001
Sex, n (%)				.41
Male	60 (81)	15 (75)	45 (83)	
Female	14 (19)	5 (25)	9 (17)	
BMI (kg/m^2^), median (range)	23.7 (17.1-32.0)	22.9 (18.4-27.9)	24.0 (17.1-32.0)	.22
ECOG PS, n (%)				.0035
0	61 (82)	12 (60)	49 (91)	
1	11 (15)	6 (30)	5 (9)	
2	2 (3)	2 (10)	0 (0)	
ASA PS, n (%)				.12
1	15 (20)	1 (5)	14 (26)	
2	51 (69)	16 (80)	35 (65)	
3	8 (11)	3 (15)	5 (9)	
CCI, n (%)				.18
0	40 (54)	8 (40)	32 (60)	
1	17 (23)	5 (25)	12 (22)	
2	8 (11)	5 (35)	3 (5)	
3	5 (7)	1 (5)	4 (7)	
4	2 (2.5)	1 (5)	1 (2)	
5	2 (2.5)	0 (0)	2 (4)	
Smoking, n (%)				.98
No	11 (15)	3 (15)	8 (15)	
Yes	63 (85)	17 (85)	46 (85)	
Abdominal surgery, n (%)				.27
No	48 (65)	11 (55)	37 (69)	
Yes	26 (35)	9 (45)	17 (31)	
Abdominal radiotherapy, n (%)				.54
No	73 (99)	20 (100)	53 (98)	
Yes	1 (1)	0 (0)	1 (2)	
Clinical T stage, n (%)				.57
a or 1 or is	25 (34)	8 (40)	17 (31)	
2	30 (41)	6 (30)	24 (44)	
3	12 (16)	3 (15)	9 (17)	
4	7 (9)	3 (15)	4 (8)	
Clinical N stage, n (%)				.012
0	64 (87)	14 (70)	50 (92)	
1	8 (11)	6 (30)	2 (4)	
2	1 (1)	0 (0)	1 (2)	
3	1 (1)	0 (0)	1 (2)	
NAC, n (%)				.11
No	37 (50)	13 (65)	24 (44)	
Yes	37 (50)	7 (35)	30 (56)	
Preoperative anemia,^‡^ n (%)				.81
No	20 (27)	5 (25)	15 (28)	
Yes	54 (73)	15 (75)	39 (72)	
Preoperative renal dysfunction,^§^ n (%)				.92
No	33 (45)	9 (45)	24 (44)	
Yes	41 (55)	11 (55)	30 (56)	
Urinary derivation method, n (%)				.091
Ileal conduit	50 (68)	16 (80)	34 (63)	
Ileal neobladder	11 (15)	0 (0)	11 (20)	
Ureterocutaneostomy	13 (17)	4 (20)	9 (17)	
Operating time (minutes), median (range)	406 (275-578)	377 (303-482)	421 (275-678)	.0025
EBL (g), median (range)	287 (17-2,300)	221 (17-964)	380 (62-2,300)	.052
Perioperative blood transfusion, n (%)				.81
No	54 (73)	15 (75)	39 (28)	
Yes	20 (27)	5 (25)	15 (28)	
Pathological T stage, n (%)				.82
0	17 (23)	5 (25)	12 (22)	
a or 1 or is	24 (32)	5 (25)	19 (35)	
2	9 (12)	2 (10)	7 (13)	
3	11 (15)	3 (15)	8 (15)	
4	13 (18)	5 (25)	8 (15)	
Number of LNs dissected (n), median (range)	16 (0-54)	14 (0-29)	16 (0-54)	.60
Pathological N stage, n (%)				.79
X	2 (3)	1 (5)	1 (2)	
0	65 (88)	18 (90)	47 (87)	
1	2 (3)	0 (0)	2 (4)	
2	4 (5)	1 (5)	3 (5)	
3	1 (1)	0 (0)	1 (2)	

BMI, body mass index; ECOG PS, Eastern Cooperative Oncology Group performance status; ASA PS, American Society of Anesthesiology physical status; CCI, Charlson comorbidity index; NAC, neoadjuvant chemotherapy; EBL, estimated blood loss; LN, lymph node.

*Elderly are patients aged ≥80 years.

^†^Non-elderly are patients aged <80 years.

^‡^Anemia is defined as hemoglobin levels of <13.5 and <12.0 g/dL in males and females, respectively.^11^

^§^Renal dysfunction is defined as an estimated glomerular filtration rate of <60 mL/min/1.73 m^2^.^12^

**Table 2.** Perioperative Complications (All Grades in the Clavien–Dindo Classification)

Variable	N (%)
Perioperative complications	
No	20 (27)
Yes	54 (73)
Highest grade I*	4 (5)
Highest grade II*	35 (47)
Highest grade III*	15 (20)
Highest grade IV*	0 (0)
Highest grade V*	0 (0)
Categories and type of complications	
Genitourinary PC	24 (32)†
UTI	16 (21)
Ureteral stenosis	3 (4)
UTI + ureteral stenosis	2 (3)
Epididymitis	2 (3)
Anastomotic leak of urinary tract	1 (1)
Gastrointestinal tract-related PC	20 (27)
Ileus	16 (22)
Enteritis	2 (3)
Constipation	1 (1)
Vomiting	1 (1)
Bleeding-related PC	16 (22)
Transfusion	15 (20)
Hematoma	1 (1)
Wound-related PC	6 (8)
SSI	4 (5)
Abdominal wall hernia	2 (3)
Intraoperative complication	5 (7)
Cardiovascular-related PC	1 (1)
Peritonitis	1 (1)
Allergy-related PC	1 (1)

N, number of patients; PC, postoperative complication; UTI, urinary tract infection; SSI, surgical site infection.

^*^The Clavien–Dindo classification.

^†^Two of the patients who developed ureteral stricture also had urinary tract infection.

**Table 3.** Perioperative Complications and Postoperative Rehospitalization Rates Within 90 Days Postoperatively According to Age and Comorbidity

	Patients	Any Grade of Complications*		Grade ≥IIIa^*^		Grade <IIIa^*^		Rehospitalization^†^	
		N (%)	*P*	N (%)	*P*	N (%)	*P*	N (%)	*P*
All patients	74	54 (73)‡		15 (20)		51 (69)		15 (20)	
Age			.81		.97		.49		.18
Non-elderly^§^	54	39 (72)		11 (20)		36 (67)		13 (24)	
Elderly^||^	20	15 (75)		4 (20)		15 (75)		2 (10)	
CCI score			.051		.87		.16		.54
0-2	65	45 (69)		13 (20)		43 (66)		11 (17)	
≥3	9	9 (100)		2 (22)		8 (89)		4 (44)	

N, number of patients; CCI, Charlson comorbidity index.

^*^ The Clavien–Dindo classification.

^†^The postoperative rehospitalization rate within 90 days postoperatively.

^‡^Twelve (16%) patients developed both grade ≥IIIa and <IIIa complications.

^§^Non-elderly are patients aged <80 years.

^||^Elderly are patients aged ≥80 years.

**Table 4. t4-tju-48-5-353:** Organ System-Based Perioperative Complications of All Grades in the Clavien–Dindo Classification According to Age and Comorbidity

	Genitourinary PC		Gastrointestinal Tract-Related PC		Bleeding-Related PC		Wound-Related PC	
	N (%)	*P*	N (%)	*P*	N (%)	*P*	N (%)	*P*
All patients	24 (32)		20 (27)		16 (22)		6 (8)	
Age		.41		.044		.28		.71
Non-elderly^*^	16 (30)		18 (33)		10 (19)		4 (7)	
Elderly^†^	8 (40)		2 (10)		6 (30)		2 (10)	
CCI score		.11		.039		.36		.72
0-2	19 (29)		15 (23)		13 (20)		5 (8)	
≥3	5 (56)		5 (56)		3 (33)		1 (11)	
	Intraoperative Complication		Cardiovascular-Related PC		Peritonitis		Allergy-Related PC	
	N (%)	*P*	N (%)	*P*	N (%)	*P*	N (%)	*P*
All patients	5 (7)		1 (1)		1 (1)		1 (1)	
Age		.71		.54		.098		.54
Non-elderly^*^	4 (7)		1 (2)		0 (0)		1 (2)	
Elderly^†^	1 (5)		0 (0)		1 (5)		0 (0)	
CCI score		.57		.0068		.70		.70
0-2	4 (6)		0 (0)		1 (2)		1 (2)	
≥3	1 (11)		1 (11)		0 (0)		0 (0)	

PC, perioperative complication; N, number of patients; CCI, Charlson comorbidity index.

^*^Non-elderly are patients aged <80 years.

^†^Elderly are patients aged ≥80 years.

**Table 5.** Perioperative Complications of Grade ≥IIIa in the Clavien–Dindo Classification According to Age and Comorbidity

	Intraoperative Complication		Ileus		Ureteral Stenosis		Abdominal Wall Hernia		Cardiovascular-Related PC	
	N (%)	*P*	N (%)	*P*	N (%)	*P*	N (%)	*P*	N (%)	*P*
All patients	5 (7)		4 (5)		3 (4)		2 (3)		1 (1)	
Age		.71		.92		.80		.45		.54
Non-elderly^*^	4 (7)		3 (6)		2 (4)		1 (2)		1 (2)	
Elderly^†^	1 (5)		1 (5)		1 (5)		1 (5)		0 (0)	
CCI score		.57		.44		.51		.59		.0068
0-2	4 (6)		4 (6)		3 (5)		2 (3)		0 (0)	
≥3	1 (11)		0 (0)		0 (0)		0 (0)		1 (11)	

PC, perioperative complication; N, number of patients; CCI, Charlson comorbidity index.

^*^Non-elderly are patients aged <80 years.

^†^Elderly are patients aged ≥80 years.

**Table 6.** Hypothesis Testing for the Difference in the Population Proportions Between Age and Comorbidity and Perioperative Complications and Postoperative Rehospitalization Rates Within 90 Days Postoperatively

	Age				CCI Score			
Variable	Non-Elderly^*^ (%)	Elderly^†^ (%)	Difference (95% CI)	*P*	0-2 (%)	≥3 (%)	Difference (95% CI)	*P*
Any grade^‡^ of complications	72	75	0.027 (−0.20 to 0.23)	.90	69	100	0.30 (0.019-0.42)	.031
Genitourinary PC	30	40	0.10 (−0.13 to 0.34)	.38	29	56	0.26 (−0.067 to 0.56)	0.12
Gastrointestinal tract-related PC	33	10	−0.23 (−0.39 to −0.013)	.035	23	56	0.32 (−0.0048 to 0.61)	.053
Bleeding-related PC	19	30	0.11 (−0.098 to 0.34)	.27	20	33	0.13 (−0.14 to 0.45)	.31
Wound-related PC	7	10	0.025 (−0.11 to 0.20)	.56	8	11	0.034 (−0.14 to 0.33)	.44
Intraoperative complication	7	5	−0.024 (−0.13 to 0.140	.98	6	11	0.049 (−0.12 to 0.34)	.37
Cardiovascular-related PC	2	0	−0.018 (−0.089 to 0.10)	.84	0	11	0.11 (−0.062 to 0.39)	.15
Peritonitis	0	5	0.050 (−0.053 to 0.19)	.25	2	0	−0.015 (−0.11 to 0.23)	.50
Allergy-related PC	2	0	−0.018 (−0.089 to 0.10)	.84	2	0	−0.015 (−0.11 to 0.23)	.49
Grade ≥IIIa^‡^	20	20	−0.0037 (−0.19 to 0.21)	.90	20	22	0.022 (−0.21 to 0.34)	.65
Intraoperative complication	7	5	−0.024 (−0.13 to 0.140	.98	6	11	0.049 (−0.12 to 0.34)	.37
Ileus	6	5	−0.0055 (−0.11 to 0.15)	.78	6	0	−0.061 (−0.16 to 0.19)	.86
Ureteral stenosis	4	5	0.012 (−0.096 to 0.17)	.58	5	0	−0.046 (−0.14 to 0.21)	.73
Abdominal wall hernia	2	5	0.031 (−0.074 to 0.18)	.40	3	0	−0.030 (−0.13 to 0.22)	.60
Cardiovascular-related PC	2	0	−0.018 (−0.089 to 0.10)	.84	0	11	0.11 (−0.062 to 0.39)	.15
Grade <IIIa^‡^	67	75	0.083 (−0.15 to 0.29)	.55	66	89	0.22 (−0.093 to 0.41)	.21
Rehospitalization^§^	24	10	−0.14 (−0.29 to 0.069)	.22	17	44	0.27 (−0.032 to 0.58)	.079

CCI, Charlson comorbidity index; PC, perioperative complication.

^*^Non-elderly are patients aged <80 years.

^†^Elderly are patients aged ≥80 years.

^‡^The Clavien–Dindo classification,

^§^Postoperative rehospitalization rate within 90 days postoperatively.

**Supplementary Table 1.** Perioperative complications and postoperative rehospitalization rates within 90 days postoperatively between intracorporeal urinary diversion and extracorporeal urinary diversion

	Patients	Elderly*				CCI ≥3			
		N (%)		*P*-value		N (%)		*P*-value	
All patients	50	34 (68)				6 (12)			
ICUD	33	19 (58)		.027		5 (15)		.33	
ECUD	17	15 (88)				1 (6)			
	Patients	Any grade of complications†		Grade ≥IIIa†		Grade <IIIa†		Rehospitalization‡	
		N (%)	*P*-value	N (%)	*P*-value	N (%)	*P*-value	N (%)	*P*-value
All patients	50	38 (76)		12 (24)		36 (72)		8 (16)	
ICUD	33	26 (79)	.52	9 (27)	.45	25 (76)	.40	4 (12)	.29
ECUD	17	12 (71)		3 (18)		11 (65)		4 (23)	

N, number of patients; CCI, Charlson comorbidity index; ICUD, intracorporeal urinary diversion; ECUD, extracorporeal urinary diversion

* Elderly are patients aged ≥80 years.

† The Clavien–Dindo classification

‡ The postoperative rehospitalization rate within 90 days postoperatively

**Supplementary Table 2.** Organ system-based perioperative complications of all grades in the Clavien–Dindo classification between intracorporeal urinary diversion and extracorporeal urinary diversion

	Genitourinary PC		Gastrointestinal tract-related PC		Bleeding-related PC		Wound-related PC	
	N (%)	*P*-value	N (%)	*P*-value	N (%)	*P*-value	N (%)	*P*-value
All patients	20 (40)		24 (28)		12 (24)		3 (6)	
ICUD	16 (48)	.088	11 (33)	.24	5 (15)	.041	2 (6)	.97
ECUD	4 (24)		3 (18)		7 (41)		1 (6)	
	Intraoperative complication		Cardiovascular-related PC		Peritonitis		**Allergy-related PC**	
	N (%)	*P*-value	N (%)	*P*-value	N (%)	*P*-value	**N (%)**	*P* **-value**
All patients	4 (8)		1 (2)		1 (2)		0 (0)	
ICUD	2 (6)	.48	1 (100)	.46	1 (100)	.46	0 (0)	N/A
ECUD	2 (12)		0 (0)		0 (0)		0 (0)	

PC, perioperative complication; N, number of patients; ICUD, ntracorporeal urinary diversion; ECUD, extracorporeal urinary diversion

**Supplementary Table 3.** Perioperative complications of grade ≥IIIa in the Clavien–Dindo classification between intracorporeal urinary diversion and extracorporeal urinary diversion

	Intraoperative complication		Ileus		Ureteral stenosis		Abdominal wall hernia		Cardiovascular-related PC	
	N (%)	*P*-value	N (%)	*P*-value	N (%)	*P*-value	N (%)	*P*-value	N (%)	*P*-value
All patients	4 (8)		4 (8)		3 (6)		0 (0)		1 (2)	
ICUD	2 (6)	.48	3 (9)	.69	3 (9)	.19	0 (0)	N/A	1 (100)	.46
ECUD	2 (12)		1 (6)		0 (0)		0 (0)		0 (0)	

PC, perioperative complication; N, number of patients; ICUD, ntracorporeal urinary diversion; ECUD, extracorporeal urinary diversion

**Supplementary Table 4.** Hypothesis testing for the difference in the population proportions between intracorporeal urinary diversion and extracorporeal urinary diversion and perioperative complications and postoperative rehospitalization rates within 90 days postoperatively

Variable	ICUD (%)	ECUD (%)	Difference (95% CI)	*p*-value
Any grade of complications*	79	71	–0.082 (−0.33 to 0.16)	0.49
Genitourinary PC	48	24	–0.24 (−0.48 to 0.035)	0.091
Gastrointestinal tract-related PC	33	18	−0.15 (−0.37 to −0.10)	0.28
Bleeding-related PC	15	41	0.26 (−0.0050 to 0.50)	0.054
Wound-related PC	6	5	–0.0017 (−0.14 to 0.18)	0.81
Intraoperative complication	6	12	0.057 (−0.11 to 0.26)	0.45
Cardiovascular-related PC	3	0	−0.030 (−0.13 to 0.12)	0.94
Peritonitis	3	0	−0.030 (−0.13 to 0.12)	0.94
Allergy-related PC	0	0	N/A	N/A
Grade ≥IIIa*	27	18	−0.096 (−0.31 to 0.16)	0.53
Intraoperative complication	6	12	0.057 (−0.11 to 0.26)	0.45
Ileus	9	6	−0.032 (−0.18 to 0.16)	0.91
Ureteral stenosis	9	0	–0.090 (−0.20 to 0.083)	0.40
Abdominal wall hernia	0	0	N/A	N/A
Cardiovascular-related PC	3	0	−0.030 (−0.13 to 0.12)	0.94
Grade <IIIa*	76	65	–0.11 (−0.37 to 0.14)	0.40
**Rehospitalization†**	12	24	−0.11 (−0.10 to 0.34)	0.30

ICUD, intracorporeal urinary diversion; ECUD, extracorporeal urinary diversion; CI, confidence interval; PC, perioperative complication

* The Clavien–Dindo classification

† The postoperative rehospitalization rate within 90 days postoperatively
